# Validation of two severity scores as predictors for outcome in Coronavirus Disease 2019 (COVID-19)

**DOI:** 10.1371/journal.pone.0247488

**Published:** 2021-02-19

**Authors:** Christian Salbach, Matthias Mueller-Hennessen, Moritz Biener, Kiril M. Stoyanov, Mehrshad Vafaie, Michael R. Preusch, Lars P. Kihm, Uta Merle, Paul Schnitzler, Hugo A. Katus, Evangelos Giannitsis

**Affiliations:** 1 Department of Internal Medicine III, Cardiology, University of Heidelberg, Heidelberg, Germany; 2 Department Internal Medicine I, Endocrinology, University of Heidelberg, Heidelberg, Germany; 3 Department of Internal Medicine IV, Gastroenterology, University of Heidelberg, Heidelberg, Germany; 4 Department for Infectious Diseases, Virology, University of Heidelberg, Heidelberg, Germany; BronxCare Health System, Affiliated with Icahn School of Medicine at Mount Sinai, NY, UNITED STATES

## Abstract

**Background:**

An established objective and standardized reporting of clinical severity and disease progression in COVID-19 is still not established. We validated and compared the usefulness of two classification systems reported earlier–a severity grading proposed by Siddiqi and a system from the National Australian COVID-19 guideline. Both had not been validated externally and were now tested for their ability to predict complications.

**Methods:**

In this retrospective, single-centre observational study, patients hospitalized with confirmed COVID-19 across all severity stages were enrolled. The clinical severity was graded at admission and during hospitalization. Multivariate Cox regression was used to identify independent risk factors for mortality, a composite primary (mortality, incident acute respiratory distress syndrome, incident mechanical ventilation), a secondary endpoint (mortality, incident acute myocardial injury, incident venous thrombosis, pulmonary embolism or stroke) and progression of severity grades.

**Results:**

Of 109 patients 17 died, 31 and 48 developed the primary and secondary endpoint, respectively. Worsening of the severity grade by at least one stage occurred in 27 and 28 patients, respectively. Siddiqi and Australian classification were identified as independent predictors for the primary endpoint (adjusted hazard ratio (aHR) 2.30, p<0.001 and aHR 2.08, p<0.001), for the secondary endpoint (aHR 2.12, p<0.001 and aHR 1.79, p<0.001) and mortality (aHR 2.30, p = 0.071 and aHR 1.98, p = 0.017). Both classification systems showed very good agreement regarding initial grading and good agreement regarding progression of severity stages.

**Conclusions:**

Standardized and objective severity grading is useful to unequivocally stratify patients presenting with COVID-19 for their individual risk of complications.

## Introduction

Coronavirus disease 2019 (COVID-19) is caused by severe acute respiratory syndrome coronavirus 2 (SARS-CoV-2). At present, nearly 27 million confirmed cases and 900,000 deaths have been reported globally by the World Health Organization (WHO) as of September 6, 2020 [1].

In most countries, health care capacities are limited and the admission to hospital and particularly transfer to the intensive care unit (ICU) is reserved for more severe or critical cases. Accordingly, the outcomes of patients with coronavirus infection are very variable, and rates of ICU admission among infected patients range from 3–100% [2]. Studies also showed that the prevalence of mortality among intensive care patients with coronavirus infection was very high ranging from 6–86% of admitted patients [2].

Assessment of the severity of the disease and risk assessment is complex and requires integration of numerous factors including demographic variables (older age, sex, ethnicity), comorbidities (COPD, asthma, heart failure), risk factors (cancer, diabetes, hypertension), presence of organ dysfunction (heart, kidney, liver, intestine, vascular endothelium) as indicated by elevation of representative biomarkers [3, 4]. Respiratory involvement, as indicated by typical radiological pathologies, low oxygen saturation, need for oxygen supply and high respiratory rate, have a relevant impact on disease severity and adverse outcomes [5–7]. Moreover, clinical scores such as q-SOFA, SIRS, and CRB-65 that are commonly calculated on admission were also found useful to provide incremental prognostic information in critically ill patients with COVID-19 pneumonia [8, 9].

A standardized classification of severity of COVID-19 at initial presentation and during follow up is paramount to increase effectiveness of patient disposition, the guidance of antiviral and adjunctive therapies and comparison between studies on COVID-19 patients in an international setting. Unfortunately, severity of COVID-19 infection at baseline and clinical worsening are not been reported in a standardized way and established definition criteria for categories of clinical severity are currently not available. Recently, two very similar multivariable classification systems were reported from the US and from Australia [6, 7].

The latter definition of disease severity was adapted from published definitions from China and Italy [10, 11]. Although both classification systems are convenient and require few commonly available variables that allow an unequivocal categorization in severity stages, the adoption in clinical practice is poor, presumably because the severity stages were not linked to outcome data and due to the lack of external clinical validation.

The objective of the present study is to validate both classification systems externally and compare them regarding their usefulness to categorize patients, to predict relevant COVID-19 related complications and to describe worsening of clinical severity and the prognostic consequences of stage-wise deterioration.

Therefore, we evaluated a consecutive cohort of hospitalised patients who presented with confirmed SARS-CoV-2 infection and categorized clinical severity at initial presentation and during hospitalization retrospectively according to both classification systems.

## Methods

### Study setting and population

We retrospectively analysed data from admitted SARS-CoV-2 PCR positive patients presenting in the University Hospital of Heidelberg between 28 February and 05 May 2020.

The diagnosis of SARS-CoV-2 infection required at least 1 positive real-time polymerase chain reaction (RT-PCR) result. Samples were routinely taken from oro- and nasopharynx or lower respiratory tract aspirates to optimize virus detection. Chest CT was conducted in patients with suspected or confirmed SARS-CoV-2 infection and symptoms suggesting viral pneumonia upon decision of treating physicians. A laboratory panel was obtained from all patients hospitalized (see laboratory tests). The decision to add laboratory markers was left at the discretion of the treating physicians. Patients with suspected or confirmed mild infections were either discharged to containment at home (excluded, when not readmitted) or admitted to the general isolation ward for monitoring the disease while waiting for the confirmation by RT-PCR result. The study was conducted in accordance with the 1964 Declaration of Helsinki declared and authorized by ethnic committee of University of Heidelberg (S-245/2020). All data was fully anonymized before data analysis. Informed consent was waived by local ethic committee due to the pandemic situation, retrospective design of the study and anonymized data analysis. Medical records were accessed between June 2020 and August 2020.

### Data collection

Baseline information including demographic, clinical, laboratory, and outcome data were obtained from electronic medical records. Information was collected on the oxygen saturation, respiratory rate, systolic and diastolic blood pressure, impairment of consciousness, the need for oxygen supplementation, and volume of oxygen supplementation at presentation and during follow-up. Where data on baseline values (blood pressure, oxygen saturation, respiratory rate) was not available, the mean value of corresponding severity stage was used instead. Several clinical scores including CRB-65, SIRS and q-SOFA were calculated retrospectively.

In addition, we recorded the days from the start of suspicious signs or symptoms of infection to hospital admission and the length of hospital stay. The clinical severity at presentation and during follow-up was retrospectively classified using the severity classification proposed by Siddiqi et al. and the National Australian severity classification [6, 7]. A detailed description of the severity grades and the variables required to classify patients is provided in the **S1 and S2 Tables**. Briefly, the Australian severity classification system categorizes the severity of clinical presentation into mild, moderate, severe and critical according to predefined criteria [7]. Likewise, the classification system proposed by Siddiqi et al. classifies patients into stage I, stage IIA, IIB and III [6]. Along with the severity grade at presentation, the occurrence and the magnitude of a worsening during observation as compared to the initial categorization was registered. Therefore, severity classification was re-calculated daily.

### Laboratory tests

The laboratory panel determined at presentation consisted of complete blood count, renal and liver function parameters (transaminases, gamma-glutamyl transferase, alkaline phosphatase), creatinine kinase, lactate dehydrogenase, electrolytes as well as acute phase reactant including C-reactive protein. The decision to add laboratory markers such as interleukin-6 (IL-6), D-dimer or procalcitonin was left at the discretion of the treating physicians. Planned re-testing of high-sensitivity cardiac troponin T (hs-cTnT) was performed at least each day to screen for incident myocardial damage [12, 13].

### Endpoint definition

The primary endpoint was defined as a composite of mortality, incident acute respiratory distress syndrome (defined according to Berlin definition [14]) or incident mechanical ventilation. The secondary endpoint consisted of a composite of mortality, incident acute myocardial injury (defined by raise and/or fall of hs-cTnT serum levels of at least 20% according to ESC guideline definition [13]), incident venous embolism, pulmonary embolism or stroke. Additional analyses included the individual components of the primary and secondary endpoint as well as worsening of the clinical stage based on the two different severity classification systems. Here, worsening of clinical stage was defined as any increase of the clinical severity class by at least one stage. Therefore, severity grading system was accessed daily. Patients presenting with a severe event at presentation were classified according to a severity class and patients were followed for incident complications, without overlap between prevalent and incident complications.

### Follow-up

All patients received a follow-up during hospital admission until discharge or twice negative RT-PCR tests at an interval of 24 hours on mortality and clinical endpoints during hospitalization using data from the electronic archive. We used time-to-event methods for censored observations. For all possible events, time to event was defined as the time from hospital admission until the date of event or censoring. All enrolled patients received a follow up until hospital discharge.

### Statistical analysis

Normally distributed continuous variables were expressed as mean ± standard deviation (SD), non-normally distributed continuous variables as median and its interquartile range (IQR). For univariate analyses, Student’s t test was performed for normally distributed data and Wilcoxon rank-sum test for non-normally distributed data. For multivariate analysis Kruskal-Wallis-test was used in case of non-normal distribution and One-Way ANOVA Test with Bonferroni post-test for normally distributed data. Categorical variables were expressed as n (% of covariant) and compared using the χ2-test. Kaplan-Meier estimates were used to draw the cumulative incidence curves, compared by log-rank tests. We used univariable and multivariable Cox proportional hazards models to identify relevant prognostic factors, with a time-to-event method for censored observations. Time to event was defined as the time from hospital admission until the date of event or censoring. Candidate predictors were identified in univariate analysis variables. Independent variables were characterized by a p-value <0.05. The adjusted hazard ratios (aHR) were presented with their 95% confidence intervals (95% CI) and the respective p-values. A two-sided p-value <0.05 was considered significant. For calculating the area under the receiver operating characteristic (ROC) curves (AUC) a predictor above 0.7 was considered useful, while an AUC between 0.8 and 0.9 was considered good. All statistical analyses are performed using MedCalc 11.1 (MedCalc Software bvba, Ostend, Belgium). The data underlying this article is available in the article and in its online supplementary; if not, it will be shared on reasonable request to the corresponding author.

## Results

**Fig 1:**
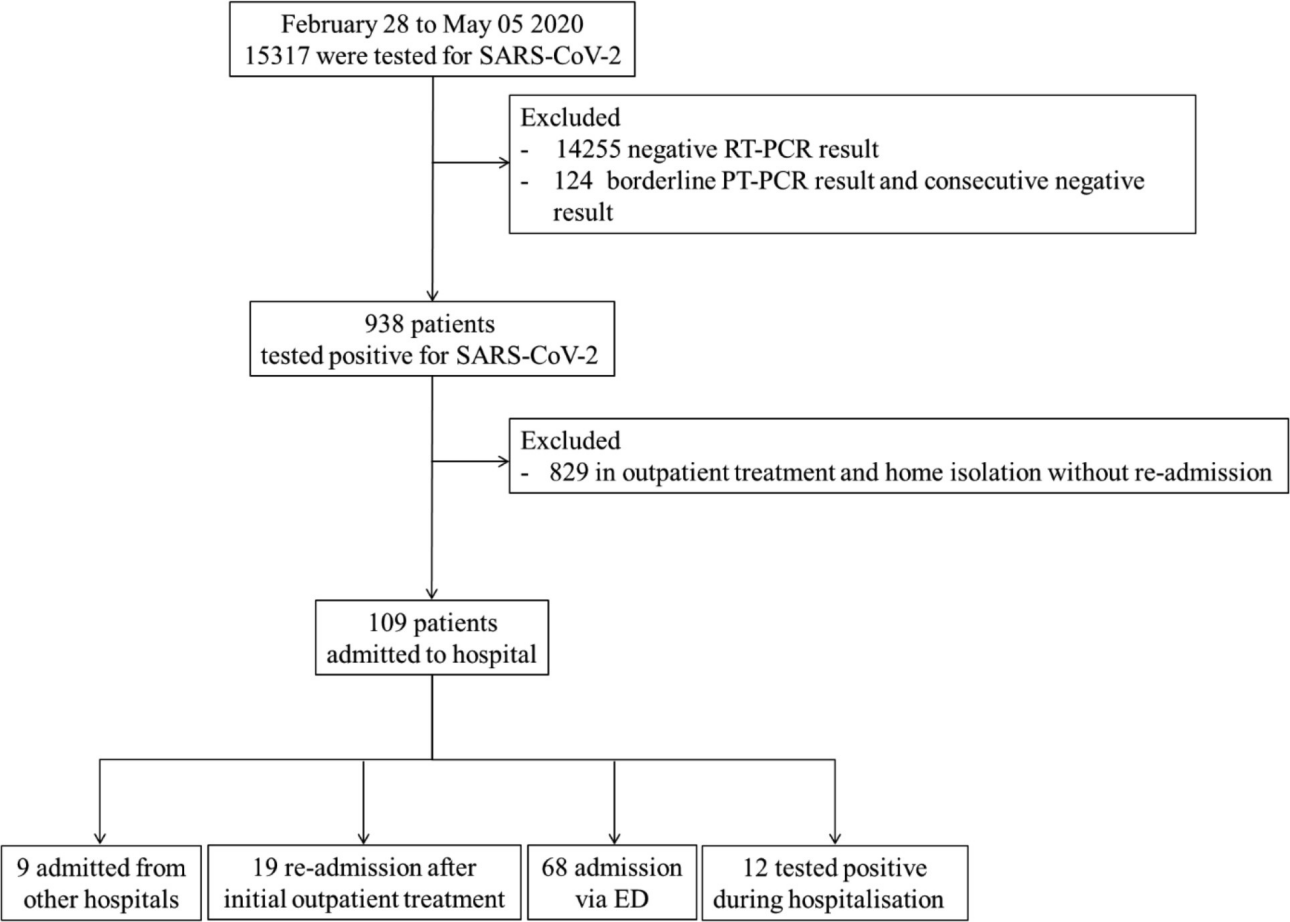
Flowchart for analysed patients. RT-PCR, real-time polymerase chain reaction; ED, emergency department.

Among 15,317 patients tested for SARS-CoV-2 at the University Hospital of Heidelberg between February 28, 2020 and May 5, 2020, diagnosis of SARS-CoV-2 infection was confirmed in 938 patients (6.1%). While 829 patients (5.4%) were excluded due to outpatient treatment, the final study population consisted of 109 patients (0.7%) hospitalized for COVID-19 comprising of 19 (17%) patients re-admitted to hospital after initial self-containment, 69 (62%) patients admitted via the emergency department (ED) or directly to ICU/general COVID-19 isolation ward, 9 (8%) patients transferred from external hospitals and 12 (11%) patients tested positive in screening tests performed routinely for patients due to admission for other reasons. A detailed overview of the patient flow can be found in **Fig 1**. Baseline characteristics according to the classification of severity stages as proposed by Siddiqi et al. are summarized in **Table 1**. Participants were aged 61.8 (SD 16.3) years, and 36 (33%) were female. Time from onset of symptoms to initial ED presentation was 7 days, (IQR 3–11) after onset of first symptoms. The prevalence of presenting symptoms varied widely across severity stages. Coughing, fever, headache, musculoskeletal complaints, a sore throat were most prevalent in Siddiqi class I and IIA, whereas diarrhoea, dyspnoea occurred in later stages, and impairment of consciousness fast almost entirely restricted to class III. An overview of laboratory findings on admission by the same severity classification is provided in **S3 Table**. There was a clear and statistically significant (P for trend <0.001) stage-wise increase of all biomarker concentrations, except for lymphocyte count, which was inversely related to clinical stages. For re-admitted patients (n = 19), which were initially treated outpatient, index-classification of Siddiqi et al. changed at re-admission to stage IIA for 4 (21%) patients, to stage IIB for 1 (5%) patient and stage III for 1 (5%) patient. A total of 13 (68%) patients remained in stage I on re-admission. Re-admission occurred with a median of 5 (SD 3.8) days after initial outpatient treatment.

**Table 1:** Baseline characteristics and analysis according to stages defined by Siddiqi et al. [6].

Variables	Stage I	Stage IIA	Stage IIB	Stage III	P Value
	(n = 46)	(n = 35)	(n = 10)	(n = 18)	
**Baseline parameters**					
**Age, mean (SD), y**	54.78 (16.3)	65.17 (15.9)	67.90 (15.9)	69.50 (10.5)	0.001
**Sex male, No (%)**	30 (65)	21 (60)	10 (100)	12 (67)	0.12
**Hospital stay, median (IQR), d**	6.5 (2–12)	9 (6–15)	8.5 (5–14)	20 (8–33)	0.014
**Onset of symptoms median (IQR), d**	7 (3–11)	7 (4–9)	13 (6–14)	7.5 (3–10)	0.30
**Admission to general ward, No (%)**	24 (52)	8 (23)	0	1 (6)	<0.001
**Admission to ICU/CCU, No (%)**	22 (48)	27 (77)	10 (100)	17 (94)	0.039
**Cardiovascular risk factors, No (%)**					
**Arterial hypertension**	16 (35)	14 (40)	6 (60)	13 (72)	0.005
**Diabetes mellitus**	4 (9)	3 (9)	2 (20)	9 (50)	<0.001
**Dyslipidaemia**	8 (17)	9 (28)	2 (20)	5 (28)	0.39
**Smoker/Ex-Smoker**	3 (7)	6 (17)	1 (10)	4 (22)	0.11
**BMI median (IQR), kg/m^2^**	25 (22–28)	27 (25–30)	27 (24–28)	29 (27–34)	0.003
**Pre-existing illnesses, No (%)**					
**Coronary artery disease**	3 (7)	5 (14)	3 (30)	6 (33)	0.003
**Chronic heart failure**	1 (2)	4 (11)	1 (10)	3 (17)	0.054
**COPD or Asthma**	2 (4)	5 (14)	0	2 (11)	0.52
**Arterial fibrillation**	4 (9)	5 (14)	3 (30)	3 (17)	0.12
**Chronic kidney disease**	5 (12)	5 (14)	0	5 (28)	0.20
**Malignant neoplasm**	6 (13)	4 (11)	1 (10)	5 (28)	0.21
**Vital parameters on admission**					
**Blood pressure systolic, median (IQR), mm Hg**	140 (134–145)	138 (130–148)	128 (116–139)	130 (118–142)	0.075
**Blood pressure diastolic, median (IQR), mm Hg**	82 (80–85)	80 (73–89)	71 (61–90)	68 (51–78)	0.003
**MAP, median (IQR), mm Hg**	101 (99–105)	102 (91–107)	87 (85–105)	86 (75–103)	0.012
**Heart rate, median (IQR), beats/min**	81 (74–87)	83 (78–91)	89 (80–100)	95 (86–130)	0.007
**Oxygen Saturation, median (IQR) %**	97 (96–98)	94 (92–97)	96 (89–98)	96 (87–99)	0.01
**Oxygen Supply, median (IQR), liters/min.**	0	0 (0–2)	4.5 (0–10)	5.0 (4–11)	<0.001
**Temperature, median (IQR), °C**	37.6 (37.4–37.6)	37.7 (37.0–38.5)	37.1 (36.3–38.4)	37.4 (36.7–38.4)	0.65
**Respiratory rate, median (IQR) breath/min.**	21 (21–21)	23 (18–27)	24 (20–28)	25 (20–31)	0.11
**Catecholamines, No (%)**	0	0	0	10 (56)	<0.001
**Mechanical ventilation, No (%)**	0	0	0	10 (56)	<0.001
**Symptoms on admission, No (%)**					
**Reduced vigilance**	3 (7)	5 (14)	2 (20)	10 (56)	<0.001
**Cough**	28 (61)	26 (74)	7 (70)	7 (62)	0.20
**Fatigue**	22 (48)	25 (71)	8 (80)	8 (44)	0.72
**Dyspnoea**	11 (24)	15 (43)	4 (40)	6 (33)	0.35
**Typical angina pectoris**	0	1 (3)	0	0	>0.99
**Atypical chest pain**	3 (7)	3 (9)	1 (10)	0	0.47
**Rhinitis**	4 (9)	1 (3)	0	0	0.091
**Sore throat**	5 (11)	2 (6)	0	1 (6)	0.31
**Limb pain**	11 (24)	7 (20)	0	1 (6)	0.036
**Headache**	9 (20)	5 (14)	0	0	0.017
**Diarrhoea**	5 (11)	6 (17)	1 (10)	2 (11)	>0.99
**Shivering**	4 (9)	2 (6)	0	0	0.16
**History of fever before admission**	30 (65)	26 (74)	6 (60)	9 (50)	0.86
**CRB-65 Score, No (%)**					
**0**	20 (44)	13 (37)	0	0	<0.001
**1–2**	26 (57)	19 (54)	10 (100)	6 (33)	0.48
**3–4**	0	3 (9)	0	12 (67)	<0.001
**qSOFA Score > 2, No (%)**	1 (2)	3 (9)	0	11 (61)	<0.001
**SIRS Score > 2, No (%)**	20 (44)	19 (54)	5 (50)	13 (72)	0.052

Values are No (% of stage), for categorical data, mean (SD) for normally distributed data and median and its (IQR) for non-normally distributed data. ICU, intensive care unit; CCU, critical care unit; BMI, body-mass-index; COPD, chronic obstructive pulmonary disease; MAP, mean arterial blood pressure; d, days; y, years; SD, standard deviation; IQR interquartile range.

### COVID-19 stages and outcomes

#### Outcomes by initial severity classification

**Fig 2:**
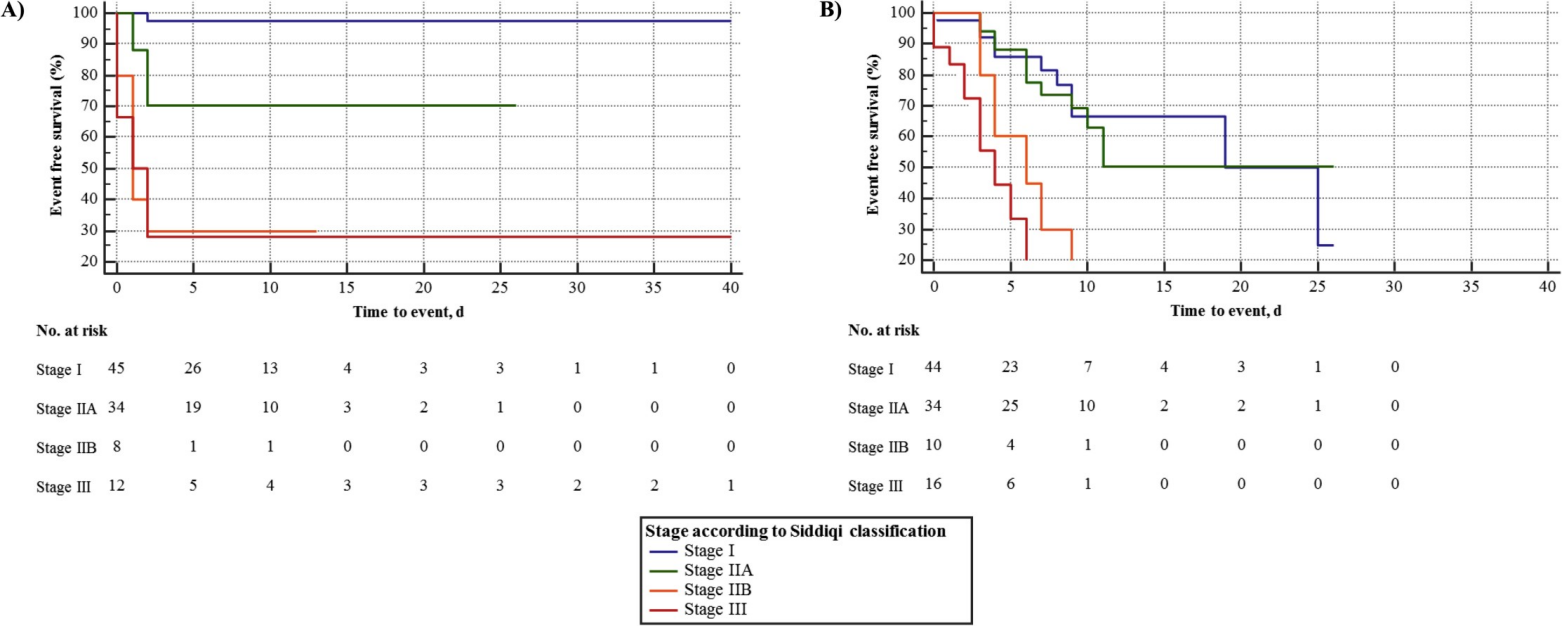
Kaplan Meier analysis for the primary endpoint and secondary endpoint by stages I-III defined by Siddiqi et al. (A) Kaplan Meier analysis for stages according to Siddiqi et al. and primary endpoint. (B) Kaplan Meier analysis for stages according to Siddiqi et al. and secondary endpoint.

A total of 17 (16%), 31 (28%) and 48 (44%) patients experienced all-cause death or developed a primary or secondary endpoint during a median observation period of 9 days (range 0 to 72 days, IQR 5–15) **Table 2**. There was a significant association between primary endpoint (P = 0.013), secondary endpoint (P<0.001) as well as mortality (P<0.001) with the clinical severity at initial presentation (**Fig 2 and S8 Table**) for the association with the classification proposed by Siddiqi et al. [6] (see supplemental material for the corresponding association with the Australian COVID-19 guideline classification [7]). During the observation period, 36 (33%) patients developed ARDS, however 10 (9%) patients were already on mechanical ventilation on admission, whereas 27 (25%) developed ARDS during hospital admission (one patient was immediately intubated during the admission process). In order to account for the impact of initial classification on outcomes, ROC analysis was performed for Siddiqi et al. and Australian COVID-19 guideline classification and primary endpoint. For classification of Siddiqi et al. on primary endpoint the ROC curve analysis showed an AUC of 0.860 (95%CI 0.78–0.92) indicating a good discrimination of Siddiqi et al. classification system on primary endpoint. For Australian COVID-19 guideline classification an ROC analysis revealed an AUC of 0.822 (95%CI 0.74–0.89) also indicating good discrimination of Australian COVID-19 guideline classification. The difference between AUC was not significant (delta AUC primary endpoint: 0.038, P = 0.199). The findings were displayed as supplemental figure including the statistical comparison of AUCs and ROC analysis of secondary endpoint (**S3 Fig**). After adjustment for other significant predictors found in univariate analysis, Cox regression was performed adjusting for age, history of arterial hypertension, COPD and diabetes, hs-cTnT on admission >99^th^ percentile, D-dimer on admission and classification according to stages defined by Siddiqi et al. or Australian guideline on admission [6, 7]. BMI, which was not significant in univariate analysis for primary endpoint (p = 0.84), secondary endpoint (p = 0.11), or mortality (p = 0.48) was not included in Cox regression model. Here, age (adjusted hazard ratio [aHR] 1.07, 95%CI 1.00–1.13, P = 0.042), COPD (aHR 4.93, 95%CI 1.05–23.18, P = 0.045) and severity stages on admission proposed by Siddiqi et al. [6] (aHR 2.30, 95%CI 1.26–4.20, P = 0.007) remained independent predictors for in-hospital death during admission. When classification according to Australian COVID-19 guideline [7] was used instead of Siddiqi et al. [6] in our Cox regression model, Australian COVID-19 guideline classification was also found to be an independent predictor for in hospital mortality (aHR 1.98; 95%CI 1.13–3.46, P = 0.007). For Cox regression of the primary endpoint, age, history of arterial hypertension, hs-cTnT on admission >99^th^ percentile, D-dimer on admission and classification on admission according to Siddiqi et al. or Australian COVID-19 guideline were adjusted [6, 7]. Here, age (aHR 1.04, 95%CI 1.00–1.08, P = 0.034) and classification on admission by Siddiqi et al. [6] (aHR 2.30 95%CI 1.57–3.35, P<0.001) were independent predictors of the primary endpoint. When classification according Australian COVID-19 guideline was used instead of classification by Siddiqi et al., Australian COVID-19 classification was found to be independent predictor for primary endpoint (aHR 2.08, 95%CI 1.48–2.92, P<0.001) [6, 7]. In Cox regression model for secondary endpoint covariates age, history of arterial hypertension, COPD, diabetes mellitus, hs-cTnT >99^th^ percentile value on admission, D-dimer on admission and classification on admission according to Siddiqi et al. or Australian COVID-19 guideline were adjusted [6, 7]. Here, hs-cTnT > 99^th^ percentile value on admission (aHR 4.35, 95%CI 1.91–9.92, P<0.001) and classification system by Siddiqi et al. (aHR 2.12, 95%CI 1.49–3.03, P<0.001) were identified to be independent predictors for secondary endpoint [6]. When classification system by Australian COVID-19 guideline was used instead of Siddiqi et al., Australian guideline classification was also found to be independent predictor for secondary endpoint (aHR 1.79, 95%CI 1.31–2.45, P<0.001) [6, 7]. Results from Cox regression analysis are shown in **Table 3**.

**Table 2:** Outcomes according to stages defined by Siddiqi et al. [6].

Variables	Stage I	Stage IIA	Stage IIB	Stage III	Total	P Value
	(n = 46)	(n = 35)	(n = 10)	(n = 18)	(n = 109)	
**Primary endpoint, No (%)**						
**Death**	0	5 (14)	3 (30)	9 (50)	17 (16)	<0.001
**Incident ARDS**	1 (2)	10 (29)	7 (70)	9 (50)	27 (25)	<0.001
**Incident mechanical ventilation**	0	4 (11)	4 (40)	6 (33)	14 (13)	<0.001
**Total**	1 (2)	10 (29)	7 (70)	13 (72)	31 (28)	<0.001
**Secondary endpoint, No (%)**						
**Incident acute myocardial injury during admission**	10 (22)	11 (31)	6 (60)	15 (83)	42 (39)	<0.001
**Venous thrombosis, pulmonary embolism or stroke**	1 (2)	1 (3)	2 (20)	5 (28)	9 (8)	<0.001
**Total**	11 (24)	12 (34)	7 (70)	18 (100)	48 (44)	<0.001
**Stage alteration during admission, No (%)**						
**Stable stage I-IIB**	34 (74)	24 (69)	5 (50)	N/A	63 (58)	<0.001
**Increase of 1 stage**	10 (22)	5 (14)	5 (50)	N/A	20 (18)	0.25
**Increase of ≥ 2 stages**	2 (4)	6 (17)	N/A	N/A	8 (7)	0.50
**Increase of > 1 stage**	12 (26)	11 (31)	5 (50)	N/A	28 (26)	0.16
**Stable stage III**	N/A	N/A	N/A	18 (100)	18 (17)	<0.001

Values are No (% of stage) for categorical data. ARDS, acute respiratory distress syndrome; N/A, data due to definition of variable not available.

**Table 3:** Significant covariates in Cox regression analysis.

Cox regression analysis for mortality			
Covariate	aHR	95% CI	P Value
**Age on admission**	11.07	1.00–1.13	0.042
**History of COPD**	4.93	1.05–23.18	0.045
**Severity stages on admission proposed by Siddiqi et al.**	2.30	1.26–4.20	0.007
**Severity stages on admission proposed by Australian COVID-19 guideline**	1.98	1.13–3.46	0.007
**Cox regression analysis for primary endpoint**			
**Age on admission**	1.04	1.00–1.08	0.034
**Severity stages on admission proposed by Siddiqi et al.**	2.30	1.57–3.35	<0.001
**Severity stages on admission proposed by Australian COVID-19 guideline**	2.08	1.48–2.92	<0.001
**Cox regression analysis for secondary endpoint**			
**Hs-cTnT > 99**^**th**^ **percentile on admission**	4.35	1.91–9.92	<0.001
**Severity stages on admission proposed by Siddiqi et al.**	2.12	1.91–3.03	<0.001
**Severity stages on admission proposed by Australian COVID-19 guideline**	1.79	1.31–2.45	<0.001

COPD, chronic obstructive pulmonary disease; hs-cTnT, high-sensitivity cardiac troponin T.

#### Impact of worsening of severity classification on outcomes

**Fig 3:**
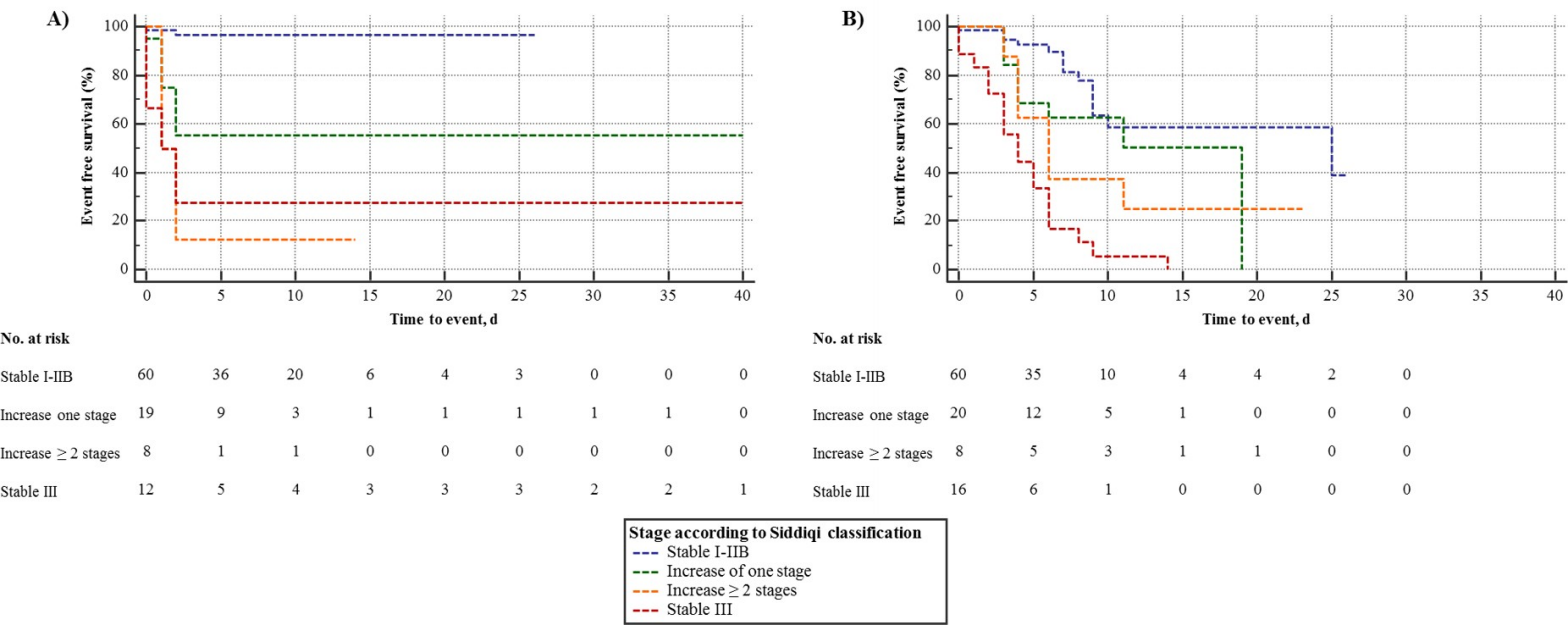
Kaplan Meier analysis of changes in clinical stages defined by Siddiqi et al. for the primary endpoint and secondary endpoint. (A) Kaplan Meier analysis of changes in clinical stages defined by Siddiqi et al. for primary endpoint. (B) Kaplan Meier analysis of changes in clinical stages defined by Siddiqi et al. for secondary endpoint. Stages were defined as stable stage I-IIA, increase of one stage, increase of at least two stages or stable stage III.

At the end of the observation period, a total of 63 (58%) patients remained stable in stage I-IIB defined by Siddiqi et al. and another 18 (17%) patients presented in stage III [6]. The remaining 28 (26%) patients demonstrated worsening of severity classes defined by Siddiqi et al. [6]. Of these, 20 (71%) progressed by one clinical stage and 8 (29%) patients by ≥ 2 stages (**Fig 3**). In a multivariate analysis, adjusted for age, history of arterial hypertension, hs-cTnT, C-reactive protein, procalcitonin and serum sodium on admission, hs-cTnT (aHR 1.01, 95%CI 1.00–1.02, P = 0.17) and C-reactive protein (aHR 1.01, 95%CI 1.00–1.01, P = 0.045), and a history of arterial hypertension (aHR 3.68 95%CI 1.27–10.69, P = 0.017) were found to predict worsening of at least one clinical stage defined by Siddiqi et al. [6]. When the Australian COVID-19 guideline classification was used instead, only C-reactive protein admission (aHR 1.01 95%CI 1.00–1.01, P = 0.029) remained an independent predictor.

#### Comparison of Australian classification and stages by Siddiqi et al

Baseline characteristics plotted by the Australian COVID-19 guideline severity classification are provided in **S4 Table**. Likewise, laboratory findings and outcome measures are shown in **S5 and S6 Tables**. In consistency with findings using the classification system proposed by Siddiqi et al., occurrence of death, the primary or secondary endpoint was found to increase with Australian severity stages (**S1 and S2 Figs**) [6]. We found a very good agreement between the two classifications, with a Cohen´s kappa of 0.804 (standard error [SE] = 0.039). The net reclassification improvement (NRI) was calculated from the classification table (**S7 Table**). A total of 24 cases of the Siddiqi et al. [6] classification were re-classified (10 higher class, 14 lower class) yielding a NRI of 0.22. The agreement between both classifications regarding severity was good, with a Cohen´s kappa of 0.810 (SE = 0.051). The reclassification table was used to compare both systems for increasing of at least one stage (**S9 and S10 Tables**). More patients were found to worsen by at least 1 stage using Siddiqi versus Australian classification (9 cases versus 4 cases). Agreement of both classification systems was good, with a Cohen’s kappa of 0.781 (SE = 0.069).

## Discussion

The precise and objective grading of the clinical severity of COVID-19 patients is paramount for correct interpretation of clinical and laboratory findings, for early and subsequent risk stratification and for guidance and timed escalation of antiviral and adjunctive therapies. So far, studies on COVID-19 continue to report unstratified data that are retrospectively grouped for post-hoc analysis by eventful or uncomplicated clinical course. Hospitalization rates, and ICU transfer do not reflect objectively the severity of disease at presentation or during hospitalization but are strongly influenced by factors such availability of hospital or ICU beds, population demographics and health care resources [15].

In an attempt to provide an objective categorization into severity grades, the Australian government introduced a severity classification that discriminates patients with mild, moderate, severe and critical severity of COVID-19 by predefined criteria [7]. In May 2020, a modified classification was proposed by Siddiqi et al., which differentiates stage I, respiratory IIA and B, and stage III [6]. Although these classifications stratify patients precisely, implementation is poor, presumably because both classifications have neither been validated independently, nor have severity classes been correlated with outcomes, so far.

Now, our study reports several important and novel findings. First, based on the panel of pre-specified clinical, radiological and laboratory findings, an accurate grading of severity at presentation and any worsening during follow-up is feasible. Both classification systems allow a consistent classification with very good agreement for initial graduation and good agreement for worsening of disease severity by at least one stage. More precisely, both classification systems showed a good agreement of comparison between stable patients in low severity, highest severity class as well as worsening of one stage and worsening of at least two stages. Second, both classification systems provide independent prognostic information for several incident complications of COVID-19 pneumonia including a composite of mortality, incident ARDS, or incident mechanical ventilation (primary endpoint), and a composite of mortality, incident acute myocardial injury, venous thrombosis, pulmonary embolism, or stroke (secondary endpoint). Third, worsening of disease severity occurs in ~25% of cases, and frequently by more than one stage, and mortality rates increase with the degree of worsening. Fourth, only few factors allow prediction of severity worsening if a classification system is implemented. In our evaluation, only C-reactive protein and hs-cTnT on admission add independent information on progression of severity by at least one stage, when the Siddiqi classification is included in the model.

### Findings in the context of previous studies

So far, there is no standardized assessment of clinical severity in COVID-19. Mild or moderate severity stages are summarized as “non-critical severities” and receive less attention until deterioration. In previous reports, a growing number of clinical and laboratory markers have been identified as independent risk predictors for hard and surrogate endpoints [4, 16–18]. However, these predictors were never tested in multivariate models that included multivariable severity stratification system.

In a large registry from China on 44,672 confirmed COVID-19 infections 81%, 14% and 5% were graded as mild, severe or critical, respectively [19]. Recently, more refined classifications propose a categorization of 5 phenotypes [20], or a more comprehensive staging like the classification proposed by the WHO [21], the Australian COVID-19 severity classification [7], and the classification proposed by Siddiqi et al. [6]. None of these classifications have been validated externally, so far.

Our findings demonstrate an in-hospital mortality rate of 15.6% that is in keeping with the mortality range of 6–15% reported in other international studies [22–24]. However, it should be emphasized that mortality rates word-wide vary widely and do not solely depend on the severity of manifestation but also on hospital resources, ICU bed and respirator capacities, numbers of available physicians, nursing and other health care professionals [25]. Furthermore, we reported rates 28.4% and 44.0% for the primary and secondary combined endpoint and rates of 24.8% and 12.8% for incident ARDS and mechanical ventilation, respectively. In other studies, rates of ARDS and mechanical ventilation vary between 9.7–68.0% depending on severity of COVID-19 cases included, and are consistent with our findings [26].

For the secondary endpoint, a comparison with the previously reported incidence of complications is challenging for two reasons. First, previous studies did not consequently monitor cardiac troponin (cTn) concentrations during hospitalization [27]. Similarly important, the presence or incidence of myocardial injury was defined by local experts [27] but was not based on the 4^th^ universal definition of myocardial infarction [28].

Regarding venous thromboembolism (VTE), we report a rate of 8.3% which is lower than previously reported 25–30% in unselected series [29, 30] and 69% in a report from two French ICUs in patients on mechanical ventilation [31]. In another multicentre French study in 1,240 patients, pulmonary embolism rates were 8.3% [32]. Differences might have been influenced by patient selection, by intensity of VTE screening and by use rates of prophylactic anticoagulation. In our cohort, patients received at least prophylactic anticoagulation from the time of hospital admission. Nevertheless, it is likely that we underestimated the true rates because patients were not routinely screened for VTE by compression ultrasonography or contrast enhanced CT angiography.

### Progression of severity

In our study, clinical severity worsened in 25.7–24.8% of cases by at least one stage depending on classification system. Of those classified by Siddiqi et al. [6], 18.3% worsened by one and 7.3% by at least two stages. Our observation is in line with a study from 3 hospitals in China, which report a progression of severity from initially mild or moderate severity to severe disease in 22.1%, to critically ill disease in 2.2% and death in 4.3% cases, totalling 28.5% of cases with progression [33]. Another study on 301 patients from China reported an overall progression rate of 21.9%, with no worsening among patients presenting initially with mild manifestation [22]. In a study on 239 patients from Italy, clinical deterioration occurred in 29.3%, including 17.2% transfers to ICU and 15.1% deaths [23]. Another Italian study on 208 patients with noncritical COVID-19 pneumonia at admission reported a clinical worsening in 63 cases (30.3%) that included death, transfer to intensive care unit, or worsening of respiratory failure [24]. The higher progression rate in the latter studies is likely explained by enrolment of patients with more severe initial severity stages than in our study, in which we enrolled patients with SARS-CoV-2 infection without signs of pneumonia, as well.

### Comparison between the Australian COVID-19 guideline classification and Siddiqi et al.

Both classifications provide an accurate stratification of severity classes as indicated by the consistently significant association between the risk for several incident events and severity classes. Prediction of the primary and secondary combined endpoint as well as mortality is similar in both classifications systems.

### Limitations

Both previously proposed classification systems were validated for the first time for feasibility and for their association with a range of important outcomes measures and complications of COVID-19. However, in our single-centre study patients were stratified retrospectively into severity classes. Before any classification system can be routinely implemented, a prospective validation using a multi-centre approach is required. Unfortunately, such a prospective multi-centre validation could not be performed at our institution–although planned–because numbers of patients with confirmed COVID-19 infection were extremely low in Germany after March 2020.

## Supporting information

S1 Fig Kaplan Meier analysis for the primary endpoint (A) and secondary endpoint (B) by stages defined by Australian COVID-19 guideline.
(TIF)

S2 Fig Kaplan Meier analysis of changes in clinical stages defined by Australian COVID-19 guideline for the primary endpoint (A) and secondary endpoint (B). Stages were defined as stable mild-severe, increase of one stage, increase of at least two stages or stable critical.
(TIF)

S3 Fig ROC analysis for stages defined by Siddiqi et al. and Australian COVID-19 guideline classification. (A) ROC curve for primary endpoint and Siddiqi et al. classification. (B) ROC curve for primary endpoint and Australian COVID-19 guideline classification. (C) ROC curve for secondary endpoint and Siddiqi et al. classification. (D) ROC curve for secondary endpoint and Australian COVID-19 guideline classification. The difference between AUC curves and classification systems was not significant: Delta AUC of primary endpoint and Siddiqi et al. (A) and Australian COVID-19 guideline classification (B): 0.038, P = 0.199. Delta AUC of secondary endpoint and Siddiqi et al. (C) and Australian COVID-19 guideline classification (D): 0.041, P = 0.189. ROC, Receiver operating characteristic; AUC, arear under the curve; CI, confidence interval.
(TIF)

S1 Table Severity classification system defined by Siddiqi et al. [6]. (DOCX)

S2 Table Severity classification system defined by Australian COVID-19 guideline classification system [7]. (DOCX)

S3 Table Laboratory findings on admission according to stages defined by Siddiqi et al. [6]. Values are mean (SD) for normally distributed data and median (IQR) for non-normally distributed data. CK, creatininase; hs-cTnT, high sensitive cardiac troponin T; LDH, lactate dehydrogenase; GOT, glutamic oxaloacetic transaminase; GPT, glutamate-pyruvate transaminase; gGT, gamma-glutamyltransferase; CRP, C-reactive protein; Hb, hemoglobin; PT. prothrombin time; INR, international normalized ratio; aPTT, activated partial thromboplastin time; PCT, procalcitonin; NT-pro BNP, n-terminal brain natriuretic peptide; IL-6, interleukin 6; WBC, white blood cells; SD, standard deviation; IQR, interquartile range.
(DOCX)

S4 Table Baseline characteristics according to according to Australian COVID-19 guideline classification [7]. Values are No (% of stage), for categorical data, mean (± SD) for normally distributed data and median and its (IQR) for non-normally distributed data. ICU, intensive care unit; CCU, critical care unit; COPD, chronic obstructive pulmonary disease; MAP, mean arterial blood pressure; d, days; y, years; SD, standard deviation; IQR interquartile range.
(DOCX)

S5 Table Laboratory findings according to Australian COVID-19 guideline classification [7]. Values are mean (± SD) for normally distributed data and median (IQR) for non-normally distributed data. CK, creatininase; hs-cTnT, high sensitive cardiac troponin T; LDH, lactate dehydrogenase; GOT, glutamic oxaloacetic transaminase; GPT, glutamate-pyruvate transaminase; gGT, gamma-glutamyltransferase; CRP, c-reactive protein; Hb, hemoglobin; PT. prothrombin time; INR, international normalized ratio; aPTT, activated partial thromboplastin time; PCT, procalcitonin; NT-pro BNP, n-terminal brain natriuretic peptide; IL-6, interleukin 6; WBC, white blood cells; SD, standard deviation; IQR, interquartile range.
(DOCX)

S6 Table Outcomes according to Australian COVID-19 guideline classification [7]. Values are No (% of stage) for categorical data. ARDS, acute respiratory distress syndrome; MI, acute myocardial injury during admission; N/A; data due to definition of variable not available.
(DOCX)

S7 Table Reclassification table between Siddiqi et al. and Australian COVID-19 guideline classification [6, 7]. Cohen´s Kappa: 0.804 (95%CI: 0.729–0.879); standard error 0.039.
(DOCX)

S8 Table Comparison of Siddiqi et al. [6] and Australian COVID-19 guideline severity classification [7] regarding prediction of outcomes, primary endpoint, secondary endpoint and mortality. (DOCX)

S9 Table Reclassification table between worsening of stages defined by Siddiqi et al. and Australian guideline [6, 7]. Cohen´s Kappa: 0.810 (95%CI 0.71–0.91); standard error 0.051.
(DOCX)

S10 Table Reclassification of worsening in clinical stages defined by Siddiqi et al. and Australian guideline [6, 7]. Cohen´s Kappa: 0.781 (95%CI: 0.645–0.917); standard error 0.069.
(DOCX)
