# Supplementary figures and images for: Validation of two severity scores as predictors for outcome in Coronavirus Disease 2019 (COVID-19)
Source: PLoS One. 2021 Feb 19;16(2):e0247488. doi: 10.1371/journal.pone.0247488 (PMC7895342; doi:10.1371/journal.pone.0247488)

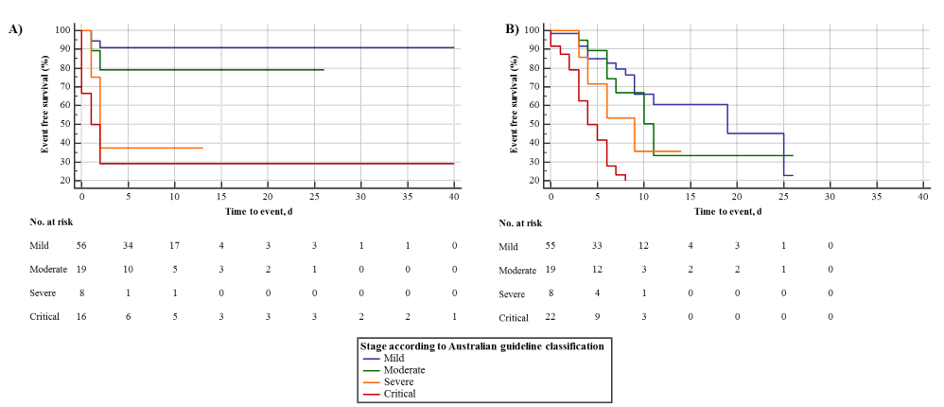

Supplement: S1 Fig — Kaplan Meier analysis for the primary endpoint (A) and secondary endpoint (B) by stages defined by Australian COVID-19 guideline. (TIF) [file pone.0247488.s001.tif]

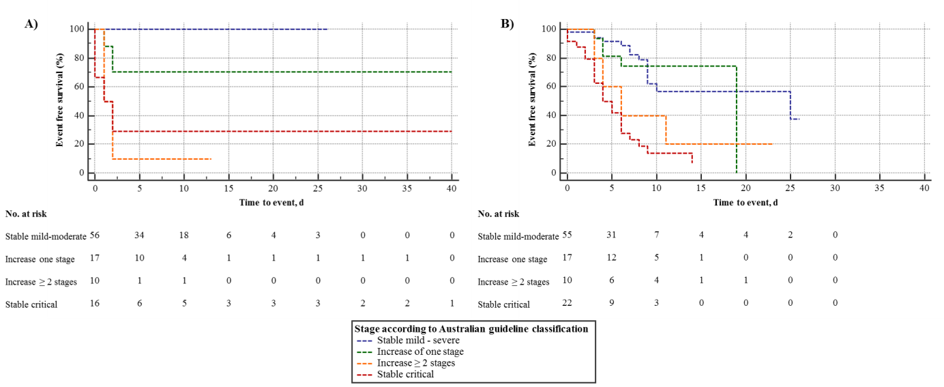

Supplement: S2 Fig — Kaplan Meier analysis of changes in clinical stages defined by Australian COVID-19 guideline for the primary endpoint (A) and secondary endpoint (B). Stages were defined as stable mild-severe, increase of one stage, increase of at least two stages or stable critical. (TIF) [file pone.0247488.s002.tif]

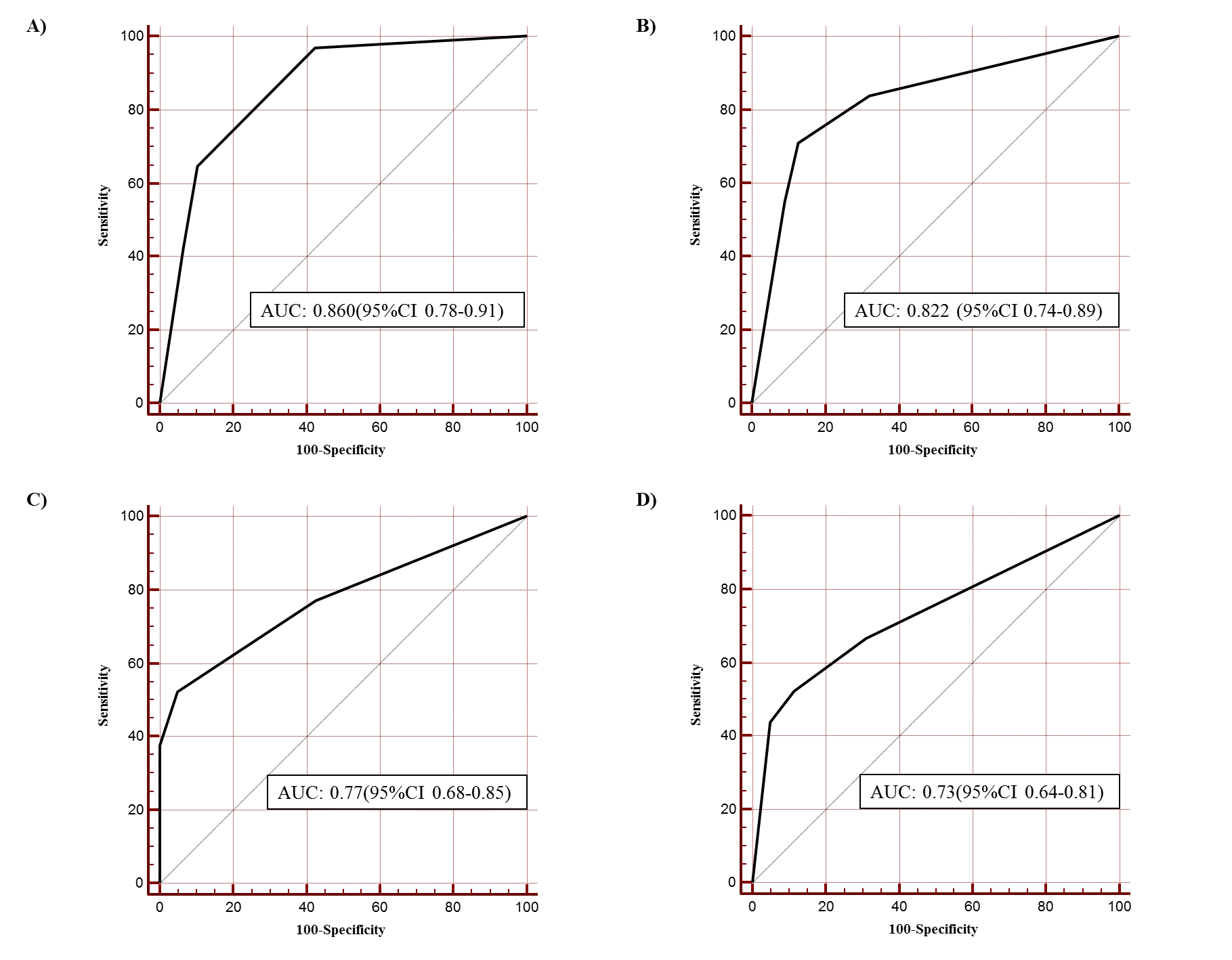

Supplement: S3 Fig — (A) ROC curve for primary endpoint and Siddiqi et al. classification. (B) ROC curve for primary endpoint and Australian COVID-19 guideline classification. (C) ROC curve for secondary endpoint and Siddiqi et al. classification. (D) ROC curve for secondary endpoint and Australian COVID-19 guideline classification. The difference between AUC curves and classification systems was not significant: Delta AUC of primary endpoint and Siddiqi et al. (A) and Australian COVID-19 guideline classification (B): 0.038, P = 0.199. Delta AUC of secondary endpoint and Siddiqi et al. (C) and Australian COVID-19 guideline classification (D): 0.041, P = 0.189. ROC, Receiver operating characteristic; AUC, arear under the curve; CI, confidence interval. (TIF) [file pone.0247488.s003.tif]
