# Supplementary material for: Validation of two severity scores as predictors for outcome in Coronavirus Disease 2019 (COVID-19)
Source: PLoS One. 2021 Feb 19;16(2):e0247488. doi: 10.1371/journal.pone.0247488 (PMC7895342; doi:10.1371/journal.pone.0247488)
Supplement: S1 Table — (DOCX) [file pone.0247488.s004.docx]

**S1 Table. Severity classification system defined by Siddiqi et al. [6].**

| **Stages** | **Symptoms** | **Oxygen Supply** |
| --- | --- | --- |
| **Stage I** | **No symptoms**  **OR**  **Nonspecific symptoms such as malaise, fever and dry caught** | **No oxygen supply is needed** |
| **Stage II A** | **Symptoms suggesting pulmonary involvement in terms of viral pneumonia (typical chest imaging revealing bilateral infiltrates or ground glass opacities)** | **Need of oxygen Supply (< 6 liters/min. via nasal prolongs)** |
| **Stage II B** | **See II A** | **Demand of oxygen supply of > 6 liters/min via nasal prolongs to maintain a saturation > 93%**  **OR**  **Saturation < 93% without oxygen supply or need of an oxygen bag**  **OR**  **High flow oxygen supply of more than 15 Liters/min**  **OR**  **manifest hypoxia (PaO2/FiO2 < 300 mmHg)** |
| **Stage III** | **Systemic hyperinflammation**  **AND**  **Systemic organ failure, manifest ARDS, manifest SIRS and shock** | **Need for mechanical ventilation to maintain adequate oxygen supply** |
