# Supplementary material for: Validation of two severity scores as predictors for outcome in Coronavirus Disease 2019 (COVID-19)
Source: PLoS One. 2021 Feb 19;16(2):e0247488. doi: 10.1371/journal.pone.0247488 (PMC7895342; doi:10.1371/journal.pone.0247488)
Supplement: S2 Table — (DOCX) [file pone.0247488.s005.docx]

**S2 Table. Severity classification system defined by Australian COVID-19 guideline classification system [7].**

| **Stages** | **Symptoms and Oxygen Supply** |
| --- | --- |
| **Mild** | **Absence of any clinical features suggesting a complicated course of illness, i.e. no symptoms**  **OR**  **Mild upper respiratory tract symptoms**  **OR**  **Cough, new myalgia or asthenia without new shortness of breath**  **OR**  **A reduction in oxygen saturation** |
| **Moderate** | **A stable patient presented with respiratory**  **AND/OR**  **Systemic symptoms or signs, able to maintain oxygen saturation above 92% (or > 90% for patients with chronic lung disease) with up to 4L/min oxygen via nasal prongs.**  **Clinical findings included prostration, severe asthenia, fever > 38°C or persistent cough, clinical or radiological signs of lung involvement, no clinical or laboratory indicators of clinical severity or respiratory impairment.** |
| **Severe** | **Respiratory rate ≥ 30 breaths/min**  **OR**  **oxygen saturation ≤ 92% at a rest state**  **OR**  **Arterial partial pressure of oxygen (PaO2)/ inspired oxygen fraction (FiO2) ≤ 300** |
| **Critical** | **Respiratory Failure: Occurrence of severe respiratory failure (PaO2/FiO2 ratio < 200) respiratory distress or acute respiratory distress syndrome (ARDS). This includes patients deteriorating despite advanced forms of respiratory support (NIV, HFNO)**  **OR**  **Patients requiring mechanical ventilation,**  **OR**  **Other signs of significant deterioration such as hypotension or shock, impairment of consciousness, other organ failure.** |
