# Supplementary material for: Validation of two severity scores as predictors for outcome in Coronavirus Disease 2019 (COVID-19)
Source: PLoS One. 2021 Feb 19;16(2):e0247488. doi: 10.1371/journal.pone.0247488 (PMC7895342; doi:10.1371/journal.pone.0247488)
Supplement: S3 Table — Values are mean (SD) for normally distributed data and median (IQR) for non-normally distributed data. CK, creatininase; hs-cTnT, high sensitive cardiac troponin T; LDH, lactate dehydrogenase; GOT, glutamic oxaloacetic transaminase; GPT, glutamate-pyruvate transaminase; gGT, gamma-glutamyltransferase; CRP, C-reactive protein; Hb, hemoglobin; PT. prothrombin time; INR, international normalized ratio; aPTT, activated partial thromboplastin time; PCT, procalcitonin; NT-pro BNP, n-terminal brain natriuretic peptide; IL-6, interleukin 6; WBC, white blood cells; SD, standard deviation; IQR, interquartile range. (DOCX) [file pone.0247488.s006.docx]

**S3 Table. Laboratory findings on admission according to stages defined by Siddiqi et al. [6].**

| **Variables** | **Stage I**  **(n=46)** | **Stage IIA**  **(n=35)** | **Stage IIB**  **(n=10)** | **Stage III**  **(n=18)** | **P Value** |
| --- | --- | --- | --- | --- | --- |
| Sodium, median (IQR), mmol/l, n=108 | 138 (134-140) | 136 (134-139) | 134 (132-136) | 138 (134-149) | 0.058 |
| Potassium, mean (SD), mmol/l, n=108 | 3.98 (0.41) | 4.00 (0.45) | 4.00 (0.55) | 4.51 (0.67) | 0.001 |
| Creatinine, median (IQR), mg/dl, n=108 | 0.74 (0.63-0.93) | 0.86 (0.69-1.04) | 0.91 (0.85-1.17) | 1.06 (0.63-2.42) | 0.039 |
| eGFR (CDK EPI), mean (SD), ml/min, n=108 | 95.9 (29.5) | 80.1 (26.2) | 76.6 (26.8) | 61.3 (36.7) | 0.001 |
| Urea, median (IQR), mg/dl, n=108 | 24 (17-32) | 25 (21-42) | 42 (32-54) | 52 (29-119) | 0.0002 |
| CK, median (IQR), U/l, n=101 | 69 (50-95) | 139 (79-240) | 169 (108-343) | 135 (77-707) | <0.001 |
| hs-cTnT, median (IQR), pg/ml, n=108 | 7.0 (4-15) | 11.0 (7-20) | 12.5 (7-25) | 32.5 (21-102) | <0.001 |
| LDH, median (IQR), U/l, n=104 | 289 (234-356) | 361 (297-441) | 550 (449-616) | 622 (509-810) | <0.001 |
| GOT, median (IQR), U/l, n=102 | 29 (19-36) | 39 (34-52) | 84 (61-230) | 82 (56-103) | <0.001 |
| GPT, median (IQR), U/l, n=102 | 27 (20-38) | 33 (27-40) | 50 (44-163) | 49 (32-79) | 0.002 |
| gGT, median (IQR), U/L n=101 | 35 (18-83) | 58 (36-107) | 52 (42-72) | 84 (34-122) | 0.046 |
| Bilirubin, median (IQR), mg/dl n=96 | 0.46 (0.40-0.60) | 0.60 (0.40-0.70) | 0.85 (0.50-1.10) | 0.70 (0.38-1.03) | 0.015 |
| CRP, median (IQR), mg/l, n=107 | 24 (8-69) | 57 (22-133) | 139 (127-186) | 165 (89-247) | <0.001 |
| WBC, median (IQR), cells/nl, n=108 | 5.1 (4.1-7.2) | 5.8 (4.1-8.2) | 5.8 (4.1-12.7) | 9.4 (7.7-14.2) | <0.001 |
| Neutrophils, median (IQR), cells/nl, n=104 | 3.5 (2.2-5.3) | 4.3 (2.9-6.8) | 4.9 (4.2-10.0) | 7.9 (6.7-12.8) | <0.001 |
| Lymphocyte , median (IQR), cells/nl, n=104 | 1.0 (0.8-1.4) | 0.8 (0.6-1.1) | 0.7 (0.6-0.9) | 0.7 (0.5-0.9) | 0.003 |
| Hb , mean (SD), g/dl, n=108 | 13.1 (1.8) | 13.7 (1.7) | 13.3 (2.3) | 11.4 (2.7) | 0.001 |
| Platelets, median (IQR), cells/nl, n=108 | 227 (172-294) | 204 (160-237) | 251 (189-304) | 245 (199-366) | 0.034 |
| PT, median (IQR), %, n=98 | 92 (83-102) | 94 (85-100) | 85 (61-102) | 75 (63-94) | 0.051 |
| INR, median (IQR), n=98 | 1.1 (1-0-1.1) | 1.0 (1.0-1.1) | 1.1 (1-0-1.3) | 1.1 (1.0-1.2) | 0.049 |
| aPTT, median (IQR), s, n=100 | 25 (23-27) | 25 (23-27) | 22 (22-23) | 26 (24-29) | 0.019 |
| D-dimer, median (IQR), mg/l, n=103 | 0.69 (0.38-1.14) | 0.84 (0.58-1.4) | 1.41 (0.85-3.6) | 9.7 (3.3-17.1) | <0.001 |
| PCT , median (IQR), ng/ml, n=106 | 0.06 (0.04-0.10) | 0.08 (0.05-0.16) | 0.19 (0.12-0.31) | 0.27 (0.15-1.1) | <0.001 |
| NT-pro BNP, median (IQR),ng/l, n=102 | 132 (60-282) | 175 (97-381) | 645 (233-1304) | 1312 (606-3712) | <0.001 |
| IL-6, median (IQR), pg/ml n=87 | 14.6 (4.1-24.4) | 37.7 (18.5-63.9) | 79.8 (36.0-89.8) | 123.0 (60.2-384.0) | <0.001 |

Values are mean (SD) for normally distributed data and median (IQR) for non-normally distributed data. CK, creatininase; hs-cTnT, high sensitive cardiac troponin T; LDH, lactate dehydrogenase; GOT, glutamic oxaloacetic transaminase; GPT, glutamate-pyruvate transaminase; gGT, gamma-glutamyltransferase; CRP, C-reactive protein; Hb, hemoglobin; PT. prothrombin time; INR, international normalized ratio; aPTT, activated partial thromboplastin time; PCT, procalcitonin; NT-pro BNP, n-terminal brain natriuretic peptide; IL-6, interleukin 6; WBC, white blood cells; SD, standard deviation; IQR, interquartile range
