# Supplementary material for: Validation of two severity scores as predictors for outcome in Coronavirus Disease 2019 (COVID-19)
Source: PLoS One. 2021 Feb 19;16(2):e0247488. doi: 10.1371/journal.pone.0247488 (PMC7895342; doi:10.1371/journal.pone.0247488)
Supplement: S4 Table — Values are No (% of stage), for categorical data, mean (± SD) for normally distributed data and median and its (IQR) for non-normally distributed data. ICU, intensive care unit; CCU, critical care unit; COPD, chronic obstructive pulmonary disease; MAP, mean arterial blood pressure; d, days; y, years; SD, standard deviation; IQR interquartile range. (DOCX) [file pone.0247488.s007.docx]

**S4 Table.** **Baseline characteristics according to according to Australian COVID-19 guideline classification [7].**

| **Variables** | **Mild**  **(n=58)** | **Moderate**  **(n=19)** | **Severe**  **(n=8)** | **Critical**  **(n=24)** | **P Value** |
| --- | --- | --- | --- | --- | --- |
| **Baseline parameters** | | | | | |
| **Age, mean (SD), y** | 57.14 (16.19) | 66.37 (18.43) | 62.50 (15.92) | 69.00 (11.54) | 0.011 |
| **Sex male, No (%)** | 36 (62) | 12 (63) | 8 (100) | 17 (71) | 0.18 |
| **Hospital stay, median (IQR), d** | 9 (4-13) | 7 (5-14) | 8.5 (4-14) | 13.5 (8-32) | 0.083 |
| **Onset of symptoms median (IQR), d** | 6.5 (3-11) | 7 (4-10) | 9 (7-14) | 7 (3-10) | 0.39 |
| **Admission to general ward, No (%)** | 28 (48) | 2 (11) | 2 (25) | 1 (4) | <0.001 |
| **Admission to ICU/CCU, No (%)** | 30 (52) | 17 (89) | 6(75) | 23 (96) | <0.001 |
| **Cardiovascular risk factors, No (%)** | | | | | |
| **Arterial hypertension** | 20 (33) | 9 (47) | 4 (50) | 16 (67) | 0.007 |
| **Diabetes mellitus** | 4 (7) | 3 (16) | 0 | 11 (46) | <0.001 |
| **Dyslipidaemia** | 11 (19) | 5 (26) | 2 (25) | 6 (25) | 0.51 |
| **Smoker/Ex-Smoker** | 4 (7) | 3 (16) | 2 (25) | 5 (21) | 0.052 |
|  |  |  |  |  |  |
| **BMI median (IQR), kg/m²** | 26 (22-28) | 28 (25-31) | 26 (25-27) | 28 (26-31) | 0.016 |
| **Pre-existing illnesses, No (%)** | | | | | |
| **Coronary artery disease** | 5 (9) | 3 (16) | 2 (25) | 7 (29) | 0.014 |
| **Chronic heart failure** | 4 (7) | 1 (5) | 0 | 4 (17) | 0.23 |
| **COPD or Asthma** | 3 (5) | 2 (11) | 2 (25) | 2 (8) | 0.37 |
| **Arterial fibrillation** | 6 (10) | 4 (21) | 0 | 15 (63) | 0.33 |
| **Chronic kidney disease** | 8 (14) | 2 (11) | 0 | 5 (21) | 0.60 |
| **Malignant neoplasm** | 8 (14 ) | 3 (16) | 0 | 5 (21) | 0.61 |
| **Vital parameters on admission** | | | | | |
| **Blood pressure systolic, median (IQR), mm Hg** | 140 (133-145) | 139 (119-150) | 136 (125-147) | 129 (117-141) | 0.11 |
| **Blood pressure diastolic, median (IQR), mm Hg** | 82 (80-85) | 78 (67-82) | 84 (75-90) | 70 (56-80) | 0.002 |
| **MAP, median (IQR), mm Hg** | 101 (94-107) | 97 (88-103) | 105 (91-107) | 87 (78-104) | 0.015 |
| **Heart rate, median (IQR), beats/min** | 82 (74-88) | 80 (78-90) | 87 (74-100) | 94 (86-108) | 0.008 |
| **Oxygen Saturation, median (IQR) %** | 96 (95-98) | 95 (92-99) | 95 (93-96) | 96 (88-99) | 0.14 |
| **Oxygen Supply, median (IQR), liters/min.** | 0 (0) | 2 (0-4) | 3 (0-5) | 6 (3-15) | <0.001 |
| **Temperature, median (IQR), °C** | 37.6 (37.4-37.8) | 37.8 (36.8-38.5) | 37.1 (36.5-38.3) | 37.6 (36.7-38.5) | 0.71 |
| **Respiratory rate, median (IQR) breath/min.** | 21 (20-22) | 23 (18-26) | 22 (18-28) | 27 (21-32) | 0.009 |
| **Catecholamines, No (%)** | 0 | 0 | 0 | 10 (42) | <0.001 |
| **Mechanical ventilation, No (%)** | 0 | 0 | 0 | 10 (42) | <0.001 |
| **Symptoms on admission, No (%)** | | | | | |
| **Reduced vigilance** | 4 (7) | 3 (16) | 2 (25) | 11 (46) | <0.001 |
| **Cough** | 37 (64) | 14 (74) | 6 (75) | 11 (46) | 0.21 |
| **Fatigue** | 30 (52) | 14 (74) | 7 (88) | 12 (50) | 0.73 |
| **Dyspnoea** | 15 (26) | 8 (42) | 4 (50) | 9 (38) | 0.20 |
| **Typical angina pectoris** | 1 (2) | 0 | 0 | 0 | 0.42 |
| **Atypical chest pain** | 4 (7) | 2 (11) | 0 | 1 (4) | 0.55 |
| **Rhinitis** | 5 (9) | 0 | 0 | 0 | 0.065 |
| **Sore throat** | 6 (10) | 1 (5) | 0 | 1 (4) | 0.25 |
| **Limb pain** | 13 (22) | 5 (26) | 0 | 1 (4) | 0.027 |
| **Headache** | 9 (16) | 5 (26) | 0 | 0 | 0.040 |
| **Diarrhoea** | 6 (10) | 3 (16) | 3 (38) | 2 (8) | 0.77 |
| **Nausea/Vomiting** | 6 (10) | 2 (11) | 0 | 1 (4) | 0.27 |
| **Shivering** | 4 (7) | 2 (11) | 0 | 0 | 0.027 |
| **History of fever before admission** | 38 (66) | 14 (74) | 6 (75) | 13 (54) | 0.69 |
| **CRB-65 Score, No (%)** | | | | | |
| **0** | 26 (45) | 7 (37) | 0 | 0 | <0.001 |
| **1 - 2** | 32 (55) | 10 (53) | 7 (88) | 12 (50) | 0.98 |
| **3 - 4** | 0 | 2 (11) | 1 (13) | 12 (50) | <0.001 |
| **qSOFA Score >2** | 1 (2) | 2 (11) | 1 (13) | 11 (42) | <0.001 |
| **SIRS Score >2** | 26 (45) | 10 (53) | 4 (50) | 17 (71) | 0.040 |

Values are No (% of stage), for categorical data, mean (± SD) for normally distributed data and median and its (IQR) for non-normally distributed data. ICU, intensive care unit; CCU, critical care unit; COPD, chronic obstructive pulmonary disease; MAP, mean arterial blood pressure; d, days; y, years; SD, standard deviation; IQR interquartile range.
