# Supplementary material for: Validation of two severity scores as predictors for outcome in Coronavirus Disease 2019 (COVID-19)
Source: PLoS One. 2021 Feb 19;16(2):e0247488. doi: 10.1371/journal.pone.0247488 (PMC7895342; doi:10.1371/journal.pone.0247488)
Supplement: S5 Table — Values are mean (± SD) for normally distributed data and median (IQR) for non-normally distributed data. CK, creatininase; hs-cTnT, high sensitive cardiac troponin T; LDH, lactate dehydrogenase; GOT, glutamic oxaloacetic transaminase; GPT, glutamate-pyruvate transaminase; gGT, gamma-glutamyltransferase; CRP, c-reactive protein; Hb, hemoglobin; PT. prothrombin time; INR, international normalized ratio; aPTT, activated partial thromboplastin time; PCT, procalcitonin; NT-pro BNP, n-terminal brain natriuretic peptide; IL-6, interleukin 6; WBC, white blood cells; SD, standard deviation; IQR, interquartile range. (DOCX) [file pone.0247488.s008.docx]

**S5 Table. Laboratory findings according to Australian COVID-19 guideline classification [7].**

| **Variables** | **Mild**  **(n=58)** | **Moderate**  **(n=19)** | **Severe**  **(n=8)** | **Critical**  **(n=24)** | **P Value** |
| --- | --- | --- | --- | --- | --- |
| **Sodium, median (IQR), mmol/l, n=108** | 137 (134-139) | 136 (132-139) | 134 (131-139) | 137 (134-148) | 0.34 |
| **Potassium, median (IQR), mmol/l, n=108** | 4.02 (3.76-4.33) | 3.86 (3.53-4.25) | 4.14 (4.04-4.19) | 4.21 (3.80-4.97) | 0.18 |
| **Creatinine, median (IQR), mg/dl, n=108** | 0.77 (0.60-1.02) | 0.86 (0.70-1.00) | 0.91 (0.73-1.04) | 1-00 (0.67-1.49) | 0.11 |
| **eGFR (CDK EPI), mean (SD), ml/min, n=108** | 90.5 (32.2) | 82.9 (22.3) | 87-2 (22.0) | 65.0 (33.7) | 0.010 |
| **Urea, median (IQR), mg/dl, n=108** | 25 (17-34) | 25 (20-37) | 28 (22-46) | 50 (32-73) | <0.001 |
| **CK, median (IQR), U/l, n=101** | 70 (49-103) | 190 (116-412) | 143 (103-291) | 159 (87-670) | <0.001 |
| **hs-cTnT, median (IQR), pg/ml, n=108** | 8 (4-15) | 11 (7-21) | 7 (6-12) | 27 (16-79) | <0.001 |
| **LDH, median (IQR), U/l, n=104** | 296 (248-367) | 369 (312-456) | 404 (313-628) | 577 (474-656) | <0.001 |
| **GOT, median (IQR), U/l, n=102** | 31 (23-39) | 43 (34-67) | 42 (29-146) | 76 (55-107) | <0.001 |
| **GPT, median (IQR), U/l, n=102** | 28 (20-38) | 37 (28-49) | 39 (25-50) | 49 (30-92) | 0.014 |
| **gGT, median (IQR), U/L n=101** | 37 (21-88) | 57 (40-107) | 44 (29-70) | 70 (35-110) | 0.31 |
| **Bilirubin, median (IQR), mg/dl n=96** | 0.50 (0.40-0.60) | 0.60 (0.40-0.73) | 0.55 (0.50-1.05) | 0.70 (0.40-1.10) | 0.080 |
| **CRP, median (IQR), mg/l, n=107** | 39 (11-77) | 44 (19-134) | 136 (112-166) | 164 (106-226) | <0.001 |
| **WBC, median (IQR), cells/nl, n=108** | 5.3 (3.9-7.4) | 4.9 (4.1-8.9) | 5.4 (4.7-10.5) | 8.9 (6.9-13.9) | <0.001 |
| **Neutrophils, median (IQR), cells/nl, n=104** | 3.8 (2.4-6.0) | 3.7 (2.9-7.1) | 4.4 (3.6-8.0) | 7.6 (5.8-12.5) | <0.001 |
| **Lymphocyte , median (IQR), cells/nl, n=104** | 1.0 (0.7-1.2) | 0.8 (0.7-1.2) | 0.6 (0.6-1.3) | 0.8 (0.5-0.9) | 0.065 |
| **Hb , mean (SD), g/dl, n=108** | 13.0 (1.8) | 13.7 (1.8) | 14.8 (1.2) | 11.8 (2.6) | 0.002 |
| **Platelets, median (IQR), cells/nl, n=108** | 215 (173-275) | 174 (144-251) | 237 (176-253) | 253 (206-335) | 0.058 |
| **PT, median (IQR), %, n=98** | 92 (83-102) | 94 (85-100) | 92 (74-102) | 78 (62-97) | 0.098 |
| **INR, median (IQR), n=98** | 1.1 (1-0-1.1) | 1.0 (1.0-1.1) | 1.1 (1.0-1.1) | 1.1 (1.0-1.2) | 0.10 |
| **aPTT, median (IQR), s, n=100** | 24.9 (23.1-26.6) | 23.9 (23.4-27.1) | 22.5 (22.4-24.6) | 25 (23-5-27.9) | 0.20 |
| **D-dimer, median (IQR), mg/l, n=103** | 0.82 (0.41-1.20) | 0.83 (0.58-1.70) | 0.89 (0.60-3.82) | 5.20 (1.18-16.04) | <0.001 |
| **PCT , median (IQR), ng/ml, n=106** | 0.06 (0.05-0.10) | 0.08 (0.06-2.43) | 0.11 (0.08-0.16) | 0.27 (0.17-0.96) | <0.001 |
| **NT-pro BNP, median (IQR),ng/l, n=102** | 136 (75-308) | 175 (75-324) | 210 (107-360) | 947 (451-3238) | <0.001 |
| **IL-6, median (IQR), pg/ml n=87** | 14.9 (8.9-34.4) | 33.8 (20.4-70.4) | 79.4 (36.2-80.3) | 88.9 (51.4-344.0) | <0.001 |

Values are mean (± SD) for normally distributed data and median (IQR) for non-normally distributed data. CK, creatininase; hs-cTnT, high sensitive cardiac troponin T; LDH, lactate dehydrogenase; GOT, glutamic oxaloacetic transaminase; GPT, glutamate-pyruvate transaminase; gGT, gamma-glutamyltransferase; CRP, c-reactive protein; Hb, hemoglobin; PT. prothrombin time; INR, international normalized ratio; aPTT, activated partial thromboplastin time; PCT, procalcitonin; NT-pro BNP, n-terminal brain natriuretic peptide; IL-6, interleukin 6; WBC, white blood cells; SD, standard deviation; IQR, interquartile range.
