# Supplementary material for: Validation of two severity scores as predictors for outcome in Coronavirus Disease 2019 (COVID-19)
Source: PLoS One. 2021 Feb 19;16(2):e0247488. doi: 10.1371/journal.pone.0247488 (PMC7895342; doi:10.1371/journal.pone.0247488)
Supplement: S6 Table — Values are No (% of stage) for categorical data. ARDS, acute respiratory distress syndrome; MI, acute myocardial injury during admission; N/A; data due to definition of variable not available. (DOCX) [file pone.0247488.s009.docx]

**S6** **Table. Outcomes according to Australian COVID-19 guideline classification [7].**

| **Variables** | **Mild**  **(n=58)** | **Moderate**  **(n=19)** | **Severe**  **(n=8)** | **Critical**  **(n=24)** | **Total**  **(n=109)** | **P Value** |
| --- | --- | --- | --- | --- | --- | --- |
| **Primary endpoint,** **No (%)** | | | | | | |
| **Death** | 1 (2) | 4 (21) | 2 (25) | 10 (42) | 17 (16) | <0.001 |
| **Incident ARDS** | 5 (9) | 4 (21) | 5 (63) | 13 (25) | 27 (25) | <0.001 |
| **Incident mechanical ventilation** | 3 (5) | 1 (5) | 2 (25) | 8 (33) | 14 (13) | <0.001 |
| **Total** | 5 (9) | 4 (21) | 5 (63) | 17 (71) | 31 (28) | <0.001 |
| **Secondary endpoint,** **No (%)** | | | | | | |
| **Incident MI** | 15 (26) | 7 (37) | 2 (25) | 18 (75) | 42 (39) | <0.001 |
| **Venous thrombosis, pulmonary embolism or stroke** | 2 (3) | 0 | 1 (13) | 6 (25) | 9 (8) | 0.001 |
| **Total** | 16 (28) | 7 (37) | 4 (50) | 21 (87) | 48 (44) | <0.001 |
| **Stage alteration during admission,** **No (%)** | | | | | | |
| **Stable (mild-severe)** | 40 (69) | 15 (79) | 3 (38) | N/A | 58 (53) | <0.001 |
| **Increase of 1 stage** | 12 (21) | 0 | 5 (63) | N/A | 17 (16) | 0.15 |
| **Increase ≥ 2 stages** | 6 (10) | 4 (21) | N/A | N/A | 10 (9) | 0.11 |
| **Increase ≥ 1 stage** | 18 (31) | 4 (21) | 5 (63) | N/A | 27 (25) | 0.023 |
| **Stable critical stage** | N/A | N/A | N/A | 24 (100) | 24 (22) | <0.001 |

Values are No (% of stage) for categorical data. ARDS, acute respiratory distress syndrome; MI, acute myocardial injury during admission; N/A; data due to definition of variable not available.
