# Supplementary material for: Validation of two severity scores as predictors for outcome in Coronavirus Disease 2019 (COVID-19)
Source: PLoS One. 2021 Feb 19;16(2):e0247488. doi: 10.1371/journal.pone.0247488 (PMC7895342; doi:10.1371/journal.pone.0247488)
Supplement: S7 Table — Cohen´s Kappa: 0.804 (95%CI: 0.729–0.879); standard error 0.039. (DOCX) [file pone.0247488.s010.docx]

**S7 Table. Reclassification table between Siddiqi et al. and Australian COVID-19 guideline classification [6,7].**

| **Australian guideline classification** | **Classification system by Siddiqi et al**. | | | | **No (%)** |
| --- | --- | --- | --- | --- | --- |
|  | **I** | **IIA** | **IIB** | **III** |  |
| **Mild** | **45** | 13 | 0 | 0 | 58 (53) |
| **Moderate** | 0 | **18** | 1 | 0 | 19 (17) |
| **Severe** | 1 | 3 | **4** | 0 | 8 (7) |
| **Critical** | 0 | 1 | 5 | **18** | 24 (22) |
| **No (%)** | 46 (42) | 35 (32) | 10 (9) | 18 (17) | 109 |

Cohen´s Kappa: 0.804 (95%CI: 0.729-0.879); standard error 0.039.
