# Supplementary material for: Validation of two severity scores as predictors for outcome in Coronavirus Disease 2019 (COVID-19)
Source: PLoS One. 2021 Feb 19;16(2):e0247488. doi: 10.1371/journal.pone.0247488 (PMC7895342; doi:10.1371/journal.pone.0247488)
Supplement: S8 Table — (DOCX) [file pone.0247488.s011.docx]

**S8 Table. Comparison of Siddiqi et al. [6] and Australian COVID-19 guideline severity classification [7] regarding prediction of outcomes, primary endpoint, secondary endpoint and mortality.**

|  | **Log-Rank Test** | **P Value** | **Adj. HR** | **95% CI** | **P Value** |
| --- | --- | --- | --- | --- | --- |
| **Primary endpoint** | | | | | |
| **Siddiqi et al.** | 49.0672 | <0.001 | 2.30 | 1.57-3.35 | <0.001 |
| **Australian guideline** | 43.4985 | <0.001 | 2.08 | 1.48-2.92 | <0.001 |
| **Secondary endpoint** | | | | | |
| **Siddiqi et al.** | 46.7076 | <0.001 | 2.12 | 1.49-3.03 | <0.001 |
| **Australian guideline** | 29.3187 | <0.001 | 1.79 | 1.31-2.45 | <0.001 |
| **Mortality** | | | | | |
| **Siddiqi et al.** | 10.8577 | 0.012 | 2.30 | 1.26-4.20 | 0.007 |
| **Australian guideline** | 10.9546 | 0.012 | 1.98 | 1.13-3.46 | 0.017 |
