# Supplementary material for: Validation of two severity scores as predictors for outcome in Coronavirus Disease 2019 (COVID-19)
Source: PLoS One. 2021 Feb 19;16(2):e0247488. doi: 10.1371/journal.pone.0247488 (PMC7895342; doi:10.1371/journal.pone.0247488)
Supplement: S9 Table — Cohen´s Kappa: 0.810 (95%CI 0.71–0.91); standard error 0.051. (DOCX) [file pone.0247488.s012.docx]

**S9 Table.** **Reclassification table between worsening of stages defined by Siddiqi et al. and Australian guideline [6,7].**

| **Classification system**  **by Australian guideline** | **Classification system by Siddiqi et al**. | | | | No. (%) |
| --- | --- | --- | --- | --- | --- |
|  | Stable (I-IIB) | Increase  of 1 stage | Increase ≥2 stages | Stable III |  |
| **Stable (mild-severe)** | **56** | 2 | 0 | 0 | 58 (53) |
| **Increase of 1 stage** | 3 | **13** | 1 | 0 | 17 (16) |
| **Increase ≥ 2 stages** | 1 | 2 | **7** | 0 | 10 (9) |
| **Stable critical** | 3 | 3 | 0 | **18** | 24 (22) |
| **No (%)** | 63 (58) | 20 (18) | 8 (7) | 18 (17) | 109 |

Cohen´s Kappa: 0.810 (95%CI 0.71 - 0.91); standard error 0.051.
