# Supplementary material for: Validation of two severity scores as predictors for outcome in Coronavirus Disease 2019 (COVID-19)
Source: PLoS One. 2021 Feb 19;16(2):e0247488. doi: 10.1371/journal.pone.0247488 (PMC7895342; doi:10.1371/journal.pone.0247488)
Supplement: S10 Table — Cohen´s Kappa: 0.781 (95%CI: 0.645–0.917); standard error 0.069. (DOCX) [file pone.0247488.s013.docx]

**S10 Table. Reclassification of worsening in clinical stages defined by Siddiqi et al. and Australian guideline [6,7].**

| **Numbers of cases with worsening by at least one stage according to Australian guideline definition** | **Number of cases with worsening by**  **at least one stage according to Siddiqi et al.** | | **No (%)** |
| --- | --- | --- | --- |
|  | no worsening | worsening |  |
| no worsening | **77** | 5 | 82 (75) |
| worsening | 4 | **23** | 27 (25) |
| **No (%)** | 81 (74) | 28 (26) | 109 |

Cohen´s Kappa: 0.781 (95%CI: 0.645 - 0.917); standard error 0.069.
